# Application of volumetric absorptive microsampling (VAMS) to measure multidimensional anti-influenza IgG antibodies by the mPlex-Flu assay

**DOI:** 10.1017/cts.2019.410

**Published:** 2019-09-26

**Authors:** Jiong Wang, Dongmei Li, Alexander Wiltse, Jason Emo, Shannon P. Hilchey, Martin S. Zand

**Affiliations:** 1 Department of Medicine, Division of Nephrology, University of Rochester Medical Center, Rochester, NY, USA; 2 Clinical and Translational Science Institute, University of Rochester Medical Center, Rochester, NY, USA

**Keywords:** Volumetric absorptive microsampling (VAMS), mPlex-Flu assay, influenza A virus antibodies, immunity assay, clinical influenza vaccine studies

## Abstract

*Introduction*: Recently, volumetric absorptive microsampling (VAMS) has been used for accurate sampling of a fixed peripheral blood volume (10 µL) on a volumetric swab, and long-term sample storage. The mPlex-Flu assay is a novel, high-throughput assay that simultaneously measures the concentration of antibodies against the hemagglutinin (HA) proteins from multiple influenza virus strains with ≤5 µL of serum. Here we describe combining these two methods to measure multidimensional anti-influenza IgG activity in whole blood samples collected by a finger stick and VAMS, with correction for serum volume based on simultaneous hemoglobin measurement. *Methods*: We compared capillary blood samples obtained from a finger stick using a VAMS device with serum samples collected by traditional phlebotomy from 20 subjects, with the influenza antibody profiles measured by the mPlex-Flu assay. *Results*: We found that results with the two sampling methods were highly correlated within subjects and across all influenza strains (mean *R*^2^ = 0.9470). Adjustment for serum volume, based on hemaglobin measurement, was used to estimate serum volume of samples and improved the accuracy. IgG measurements were stable over 3 weeks when VAMS samples were stored at room temperature or transported using a variety of shipping methods. Additionally, when volunteers performed finger-stick VAMS at-home by themselves, the comparison results of anti-HA antibody concentrations were highly consistent with sampling performed by study personnel on-site (*R*^2^ = 0.9496). *Conclusions*: This novel approach can provide a simple, accurate, and low-cost means for monitoring the IgG anti-influenza HA antibody responses in large population studies and clinical trials.

## Introduction

Both seasonal and emerging influenza virus infection are among the largest reoccurring global public health threats [1], and vaccination is the major method of prevention [2]. Flu vaccines are currently designed to elicit antibodies against hemagglutinin (HA), the most abundant glycoprotein on the viral surface [3]. Protective antibodies block the ability of HA to bind to sialic acid on target cells, or enhance viral clearance, preventing infection [4]. Measuring antibody-mediated immunity is critical to evaluate vaccine efficacy and immunity to seasonal and emerging influenza viruses. However, a major translational barrier is obtaining serum samples to measure antibody-mediated influenza immunity, which is resource-intensive, time-consuming, and expensive [5]. This limits our ability to conduct large-scale influenza vaccine clinical trials, measure population immunity, and assess the mismatch between circulating influenza strains and the seasonal influenza vaccine in real time. Solving this translational barrier would greatly improve our ability to conduct high-quality clinical trials of influenza vaccines, perform large-scale assessments of population immunity to influenza, and greatly decrease the resource intensity of clinical and translational influenza research.

Several factors contribute to this translational barrier. Most assays of antibody-mediated influenza immunity, such as hemagluttinin inhibition (HAI), enzyme-linked immunosorbent assay (ELISA), and microneutralization (MN) assays all require at the minimum 0.1–0.2 mL of serum to perform with appropriate replicates. Such quantities of serum are usually obtained by venipuncture phlebotomy performed by a healthcare professional, requiring subject travel to the research facility or collection point. After collection, blood samples require post-phlebotomy processing including serum separation, aliquotting, and storage. Thus, developing a translational research solution would require addressing two barriers: (1) developing and validating a simple method for in-the-field collection of small amounts of peripheral blood or serum, and (2) coupling microsampling to an assay measuring anti-influenza IgG that uses very small sample volumes (5–20 µL). Here we describe and validate such a system, using a combination of volumetric absorptive microsampling (VAMS) [6] coupled with a Luminex-based assay (mPlex-Flu) [7–12] to quantitatively measure IgG antibodies against 33 strains of influenza HA.

One approach to simplifying sample collection is to perform a finger or heal stick to draw a drop of blood, using a disposable lancet as is done for diabetic blood glucose monitoring. The blood drop, generally 50–200 µL, is adsorbed onto filter paper and dried. Samples are then eluted and analyzed at a later date. This microsampling dried blood spot (DBS) method was first introduced in 1963 [13]. It has been used to assess the HIV-1 antibodies in newborns, in population-based surveys for more than 25 years [14–16], and for analysis of anti-drug antibodies in FDA clinical trials. DBS is safer and simpler than venipuncture. It enables self-sampling at-home and can greatly reduce costs for clinical- or population-based studies. In addition, IgG and IgM antibodies in dried blood sample are known to be stable at room temperature for weeks and at −20°C for years. A significant drawback of DBS, however, is the high variability of sample volumes. This makes calculation of a concentration problematic and limits its use for quantitative measures of antibody abundance. In contrast, VAMS devices adsorb a consistent volume of blood from a finger stick, generally 10 or 20 µL, and have been recently used to collect samples for antibody testing in many fields (Reviewed in Ref. [6]). This new technique overcomes the issue of inconsistent blood volumes between sample blood spots in the DBS method. VAMS allows accurate and precise sampling with standard deviation ≤0.4 µL with 10 µL blood samples [17].

To address the translational barrier of measuring anti-influenza antibody-mediated immunity, we have previously developed a Luminex-based multiplex assay (mPlex-Flu assay) that can simultaneously measure absolute antibody concentrations (IgG, IgM, or IgA) against up to 50 influenza strains using ≤5 µL of serum [7,8,11,12]. The mPlex-Flu assay has a continuous linear readout over four logs, with low Type-I (false positives, specificity) and Type-II (false negatives, sensitivity) errors [10]. It provides absolute concentrations for strain-specific anti-influenza IgG antibody levels, as opposed to 8–12 discrete titer levels for other assays (e.g. HAI, MN), with extremely low inter- and intra-subject variance [9]. Notably, the mPlex-Flu assay also has a very high correlation with both standard HAI and MN assays, with several added advantages, including simultaneous measurement of absolute anti-HA IgG levels for a large number of influenza strains [7,8,11,12], greater precision of clinical trial group statistical comparisons [9,10], and a low per-sample cost.

Unlike traditional DBS sampling, VAMS also permits whole blood IgG measurements to be adjusted for serum or plasma volume. Whole blood is composed of a cellular component, primarily erythrocytes, and a noncellular component, plasma (when anticoagulant is used) or serum (when clotting is allowed to occur). The hematocrit (HCT) is the fraction of whole blood occupied by cells; IgG is only present in the noncellular serum fraction (∼45–75%). Thus IgG concentrations, as traditionally measured in plasma, (*IgG*_*s*_) will be higher than those measured in whole blood (*IgG*_*BL*_) such that [*IgG*]_*BL*_ = [*IgG*]_*S*_(1 – *HCT*) [18]. This factor could lead to systemic underestimation of anti-HA IgG levels with VAMS. HCT cannot be measured directly in a DBS or VAMS sample. However, the concentration of hemoglobin (Hgb), an iron-binding molecule contained by red blood cells, can be measured. There is a tight correlation between whole blood Hgb levels and HCT, generally *HCT*_*BL*_ = 3.0[*Hgb*]_*BL*_. While some exceptions apply (e.g. sickle cell disease, *β* thallasemia) [19], such estimates of HCT from Hgb generally have only a modest error under normal conditions [20]. As blood sample volume is known in VAMS [21], [*Hgb*]_*BL*_ can be used to estimate *IgG*_*S*_, facilitating comparison of VAMS measured anti-HA IgG concentrations with more traditional direct plasma measurement methods.

Development of a simple method to measure anti-HA IgG levels using small volume blood samples remotely collected by study subjects would greatly improve our ability to conduct more robust clinical trials, population immunity surveys, and augment current influenza surveillance efforts. For example, most influenza vaccine clinical trials have measured anti-HA IgG titers in peripheral blood samples pre-vaccination (day 0), and at days 7 and 28 post-vaccination [22], while others have collected samples at more distant time points [23]. A substantial expense in these trials is sample collection by trial personnel. For the same reasons (i.e. cost and inconvenience of phlebotomy for sample collection), large-scale surveys of population antibody-mediated immunity to influenza are rare. We are not aware of any current studies assessing IgG-mediated immunity to multiple (>30) influenza strains with over 1000 subjects. Finally, the the United States Center for Disease Control and the World Health Organization (WHO) both conduct extensive influenza virus field surveillance programs [24], collecting viral samples by nasal swab to isolate and sequence influenza strains in people with influenza-like illnesses. Yet, these programs generally do not collect serum to assess antibody-mediated influenza immunity, likely due to the cost and time needed for phlebotomy and sample processing. In these cases, a simple method to collect and analyze samples for anti-influenza IgG concentrations would decrease the barriers to multiple sample collection (cost, inconvenience, sample processing), and improve scientific knowledge of influenza immunity.

Here we describe using a combination of VAMS with the mPlex-Flu assay and Hgb measurement to quantitatively estimate serum IgG antibody concentrations against 33 strains of influenza virus HAs. Whole blood anti-influenza IgG concentrations are adjusted for serum volume using measured Hgb and used to estimate serum anti-influenza IgG to facilitate comparison with standard methods. This study validates the accuracy, reproducibility, and sample stability of this novel assay combination. Overall, we show that the combination of VAMS with the mPlex-Flu assay could be a powerful tool for large sample size analysis of multidimensional influenza antibody-mediated immunity for use in influenza vaccine and population immunity studies.

## Methods

### Human Subjects Ethics Statement

This study was approved by the Research Subjects Review Board at the University of Rochester Medical Center (RSRB approval number RSRB00070463), and informed consent was obtained from all participants. Research data were coded such that subjects could not be identified, either directly or through linked identifiers. Subject identification numbers were re-encoded for publication.

### Participants and Sample Collection

Twenty-one healthy volunteers 18–65 years of age were recruited for this study. Subjects who were taking immunosuppressive medications were excluded. All subjects had samples collected by both venous phlebotomy and VAMS (Mitra Collection Kit; Neoteryx, CA, USA). For venous phlebotomy, standard venipuncture was performed and 3–4 mL of blood was collected in a serum collection tube (BD, NJ, USA), centrifuged (3000 RPM, 4°C, 12 min), and sera were aliquotted into 100 µL cryo-vials and stored at −20°C until analysis.

### Study Design, VAMS Sample Collection and Storage

**Fig. 1.**
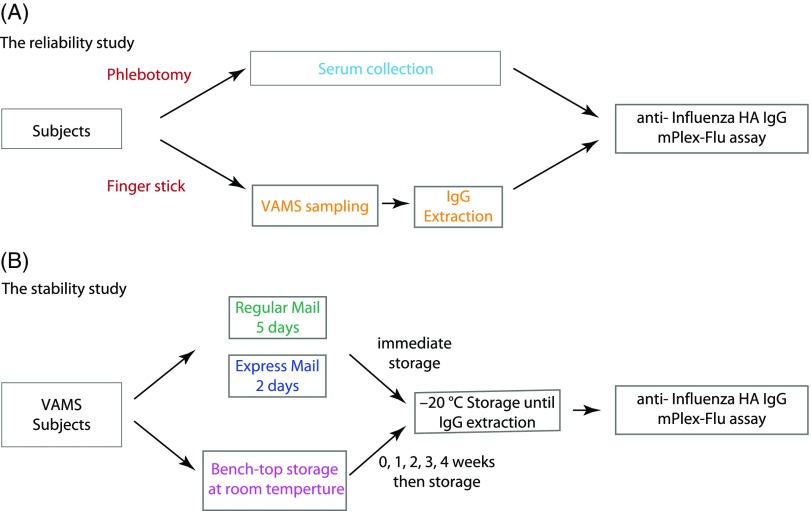
Experimental design. HA = hemagglutinin; VAMS = volumetric microsampling.

The study was designed to assess both variability between standard venipuncture for serum and VAMS sampling, and reproducibility of results when subjects performed VAMS sampling remotely after instruction. At the initial study visit, each volunteer donated one venous blood sample by phlebotomy and one VAMS blood sample by finger stick. Both samples were collected by study coordinators on-site at the University of Rochester Clinical Research Center. Study subjects were then trained to perform a finger stick with the lancet device and collect the VAMS sample. After training, one VAMS kit was sent home with the volunteer. Three days later, the volunteer self-collected a second VAMS sample and returned it in sealed packaging to the Research Center for analysis (Fig. 1).

VAMS blood samples were collected using the manufacturer’s 10 µL collection kit (Neoteryx, CA, USA). After alcohol swabbing, the lateral portion of the participant’s finger was punctured using a contact-activated lancet. Gentle pressure was applied to the finger to allow a drop of blood to collect at the skin surface. A porous, hydrophilic VAMS tip was held against the blood drop until completely filled with blood. Each tip absorbed 10 µL of blood, and two tips were present in each collection kit, for a total of 20 µL of blood per collection. Tips were allowed to dry for 1–2 h at room temperature in protective cassettes. For the stability experiment, 14 VAMS blood samples from one donor were collected at the same time. All VAMS tips were placed in separate and sealed containers with silica desiccant packets and stored at −20°C until analysis.

### Extraction of Antibodies and Hemoglobin (Hgb) from VAMS Samples

The absorbent tips from each VAMS collection kit (containing 10 µL blood sample) were soaked in 200 µL extraction buffer (PBS + 1% BSA + 0.5% Tween) in 1 mL 96-well plates (Masterblock, GBO, Austria) and shaken overnight to extract the antibodies as described previously [16]. Hgb was extracted along with IgG by this method and quantified as described below.

### mPlex-Flu Assay

The mPlex-Flu assay was performed as described previously [7,8,12]. Briefly, venous phlebotomy serum samples were diluted 1:5000 with PBS, while the 200 µL extractions from VAMS device (1:20) were further diluted 1:250, to yield a final 1:5000 dilution of the VAMS samples. For both serum and VAMS samples, 200 µL of diluted sample was used for analysis and added to a black, clear-bottom 96-well plate (Microplate, GBO, Austria). Standard serum (STD01) was made in our laboratory [7,12], and the standard curve for each influenza virus strain was generated by 1:4 serially diluting STD01 for each batch of samples. Fifty microliter of the diluted sample was added into each reaction well. All samples were run in duplicate.

The influenza HA bead panel used in this study is shown in Table 1, comprising 30 separate recombinant HAs. Fifty microliter of beads mix was added to each well of the plate as previously described [7,8,12]. Plates were incubated with gentle shaking for 2 h at room temperature and then washed (PBS + 1% Bris + 0.1% BSA). A magnet placed under the plate immobilized the beads during washes. After three washes, a goat anti-human IgG-PE secondary antibody (Southern Biotech, Cat No:2040–09) was added, and plates were incubated for another 2 h. After three more washes, beads were resuspended in drive fluid (Luminex Co., TX) and the beads were analyzed using MAGPIX Multiplex Reader (Luminex Co., TX). The calculation of IgG antibody concentration against each individual influenza virus strain rHA was performed by Bio-Plex Manager 6.2 software (Bio-Rad Co., CA).

**Table 1.** The panel of influenza virus recombinant hemagglutinins (rHAs) in mPlex-Flu assay

Influenza type	Subtype	Full strain name	Abbreviation	Genbank accession #
A	H1	A/South Carolina/1/18	SC18	AF117241.1
		A/Puerto Rico/8/1934	PR8	CY148243.1
		A/USSR/90/1977	USSR77	DQ508897.1
		A/New Caledonia/20/1999	NewCal99	DQ508889.1
		A/Texas/36/1991	Tex91	CY125100.1
		A/California/07/2009	Cali09	FJ966974.1
		A/Michigan/45/2015	Mic15	KY117023.1
	H2	A/Japan/305/1957	Jap57	L20407.1
	H3	A/Port Chalmers/1/1973	PC73	CY112249.1
		A/Hong Kong/1/1968	HK68	CY009348.1
		A/Perth/16/2009	Per09	GQ293081.1
		A/Victoria/361/2011	Vic11	KM821347
		A/Texas/50/2012	Tex12	KC892248.1
		A/Switzerland/9715293/2013	Swi13	EPI537866
		A/Hong Kong/4801/2014	HK14	EPI653201
		A/Singapore/INFIMH-16-0019/2016	Sin16	EPI1164036
	H5	A/Viet Nam/1203/2004	Viet04	EF541403
		A/Beijing/01/2003	BJ03	EF587277
	H6	A/chicken/Taiwan/67/2013	chTW13	KJ162860.1
	H7	A/mallard/Netherlands/12/2000	malNert00	EF470586
		A/rhea/North Carolina/39482/1993	rheaNC93	KF695239
		A/Shanghai/1/2013	SH13	KF021597.1
	H9	A/guinea fowl/Hong Kong/WF10/1999	gfHK99	AY206676.1
B		B/Brisbane/60/2008	B/Bris08	CY115343
		B/Massachusetts/2/2012	B/Mass12	KF752446.1
		B/Phuket/3027/2013	B/Phu13	EPI540673
Chimeric HA	Group 1 Stalk	A/Indonesia/5/05 head, A/California/07/2009 stalk	cH5/1	
		A/gf/HK/WF10/1999 head, A/California/07/2009 stalk	cH9/1	
	Group 2 Stalk	A/Indonesia/5/05 head, A/Victoria/361/2011 stalk	cH5/3	
		A/duck/Czech/1956 head, A/Shanghai/1/2013 stalk	cH4/7	

### Measurement of Hemoglobin (Hgb) and Hematocrit (HCT)

We measured Hgb concentrations using a Hemoglobin colormetric Assay Kit (Abcam, Cat No:ab234046, MA, USA). Briefly, 20 µL of blood or extracted samples were incubated with 180 µL of Hgb detector buffer at room temperature for 15 min in 96-well plates. Negative and positive controls for Hgb at concentrations of 0, 50, 100, 150, 200, and 250 mg/dL six standards were used to construct a standard curve. The absorbency at 575 nm (*OD*_575_) was measured using a Synergy Microplate reader (BioTek, VT, US). The concentration of Hgb was then calculated based on the *OD*575 of samples with the standard curve. The HCT of venous whole blood samples taken by venipuncture was measured using an AcT Diff Coulter Counter (Beckman Coulter, CA) following manufacture instructions.

### Estimation and Adjustment of the Effects of HCT on IgG Concentration in VAMS Saples

To create a standard curve to estimate serum IgG concentration, we collected heparinezed whole blood by phlebotomy (Ref No:366480, BD Vacutainer), and separated the cellular and plasma portions from each subject by centrifugation. We next created a range of HCTs, (∼10%, 20%, 30%, 40%, 50%, and 60%) by manually mixing the plasma and red blood cells in a 2-mL total volume. As the volume of red cells might vary even with accurate pipetting, we measured the HCT and Hgb using the Coulter Counter, as described above, for all control blood mixtures, and used the resulting values for further calculation. We next collected VAMS samples in triplicate from the standards. HCT and Hgb were measured for all VAMS samples, and anti-influenza HA IgG antibody concentrations were measured from the corresponding venipuncture serum samples and the VAMS sample extractions.

To obtain the estimated serum concentration ([*IgG*_*S*_]) from the VAMS blood concentration ([*IgG*_*VAMS*_]), we first built a relationship between the estimated ratio of 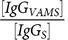 and the measured HCT values from five subjects using a generalized estimating equation (GEE) model with identity link function [25]. A compound symmetry variance–covariance matrix [26] was used to model the within-subject correlation. The GEE model gives us the following relationship between the estimated ratio of 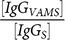 and the measured real HCT values.(1)
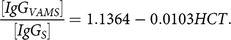



Next, we obtained the relationship between HCT values and Hgb values using measured HCT and Hgb data from five subjects through the GEE model with the identity link function and compound symmetry variance–covariance matrix:(2)
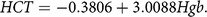
Combining equations 1 and 2 results in the following relationship between the estimated ratio of 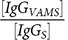 and the measured real *Hgb* values.(3)
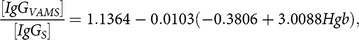
which can be used to estimate the serum anti-influenza HA IgG concentration from the hemaglobin measurement:(4)
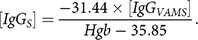



### VAMS Sample Stability Analysis

To assess the stability of VAMS samples at room temperature over time, 14 VAMS samples were collected from the same subject. An initial two VAMS samples were stored at −20°C after drying for 2 h. A further eight VAMS samples were left on the lab bench at room temperature (22–25°C, controlled, but not monitored). Two of these were then moved into 20°C storage at days 7, 14, 21, and 28 after the initial sampling. The remaining four VAMS samples were mailed back to the lab using the United States Postal Service (USPS) and 2-day overnight delivery. The samples sent by express delivery service returned in 2 days and those sent by the standard post returned in 5 days (Fig. 1). The VAMS samples were stored at −20°C upon arrival in the laboratory.

### Statistical Analysis

Spearman’s correlation coefficient [27] with the Benjamini–Hochberg multiple testing correction method [28] was used to measure the reliability of mPlex-Flu results from VAMS versus conventional venous phlebotomy samples, the reproduciblity of mPlex-Flu results from VAMS collected by volunteers at-home versus VAMS collected by study coordinators on-site, and the stability of mPlex-Flu results from VAMS samples stored at room temperature over time or after shipping. For calculation of correlation coefficients, measurements from the mPlex-Flu assay using various VAMS samples were either combined across multiple influenza virus types or separated by influenza virus type and subject.

Subject demographic differences were analyzed using the binomial exact test. Because the sample size is small and the data were not normally distributed, we used GEE models with identity link functions [29] to compare the mean measurements from the mPlex-Flu assay results obtained with VAMS versus conventional serum sampling under different room temperature storage times and shipping methods. GEE models with identity link functions were also used to build the relationship between the estimated serum concentration ([*IgG*_*S*_]) and the VAMS blood concentration ([*IgG*_*VAMS*_]. The within-subject correlations were accounted for using compound symmetry variance–covariance matrix.

Statistical analysis software SAS v9.4 (SAS Institute, Inc., Cary, NC) and R version 3.5.1 were used for all the data analysis. The significance level for all tests was set at *P* = 0.05.

## Results

### Subject Demographics

Twenty-one healthy volunteers were recruited for this study and their demographics are shown in Supplementary Table 1. More female subjects took part in this study (71%) than male (*P* = 0.0784), with majority of volunteers being Caucasian (90%; *P* = 0.002). The distribution of age groups is relatively uniform with fewer volunteers ≤20 years of age.

### mPlex-Flu Assay Results from VAMS and Serum Sampling are Highly Correlated

**Fig. 2.**
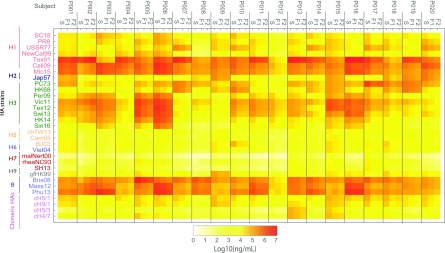
Anti-HA IgG antibody concentration against 30 influenza virus strains assessed by mPlex-Flu assay. The blood samples of 20 subjects were collected by phlebotomy serum sampling (S), VAMS sampling on-site (F1), and VAMS sampling at-home (F2) were tested by mPlex-Flu assay with a 30 influenza virus HA panel in the same 1:5,000 dilution. The IgG concentrations of samples were calculated based on a standard curve for individual virus strain generated by standard serum with Bio-Plex Manager 6.2 software. The mean concentration of duplicates are shown in the heatmap. HA = hemagglutinin; VAMS = volumetric absorptive microsampling. Influenza strain full names are provided in Table 1.

In order to compare the variability of mPlex-Flu results between capillary blood VAMS versus venous serum sampling, we measured anti-influenza IgG concentrations using the mPlex-Flu assay on simultaneous VAMS finger stick and serum from venipuncture samples (*n* = 20 subjects). The results are shown in heatmap form in Fig. 2.

**Fig. 3.**
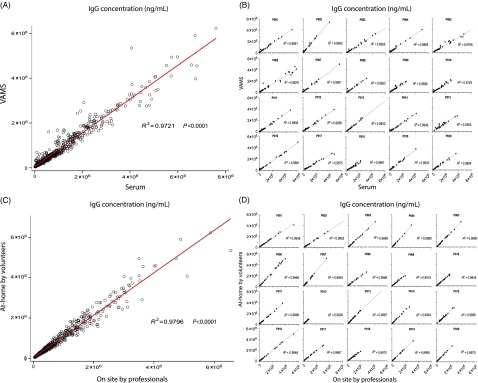
Correlation analysis. (A and B) The correlation of concentration of influenza virus IgG antibodies against 30 strains of influenza virus by mPlex-Flu assay using VAMS sampling versus venous serum sampling. (A) The overall correlation (*n* = 620); (B) the analysis separated by individual subject (*n* = 31). (C and D**)** The correlation of concentration of anti-influenza virus IgG antibodies against 30 strains of influenza virus by mPlex-Flu assay with VAMS finger stick from on-site professionals with that from volunteers at-home. (C) The overall correlation (*n* = 620). (D) The correlation of concentration of influenza virus antibodies separated by individual subject (*n* = 31). HA = hemagglutinin; VAMS = volumetric absorptive microsampling.

To compare the anti-HA IgG concentrations from mPlex-Flu in samples obtained by VAMS versus traditional phlebotomy, we used the Spearman’s correlation coefficient [27] with the Benjamini–Hockberg multiple testing correction method [28]. We found a high overall correlation of the mPlex-Flu results between the two sampling methods (*r* = 0.9721;*P* < 0.001) (Fig. 3A) with *n* = 20 subjects assayed for anti-HA IgG against 30 strains of influenza (total *n* = 620 data points).

We also found high correlations when we calculated the (*r*) values separated by individual influenza virus strains for 20 subjects. All results are listed in Table 2, with the mean *r* = 0.9470. When we calculated the correlation (*r*) values separated by individual subject for 30 influenza virus strains, we found the even higher correlation (*r*) values. The results are shown in Fig. 3B, with the mean *r* = 0.9836, which is likely due to smaller within-subject variability than between-subject variability. All the above results suggest that mPlex-Flu results from VAMS are highly correlated with that of serum sampling when assessing individual strain-specific anti-HA IgG for individual human subjects.

**Table 2.** Correlation between mPlex-Flu anti-HA IgG results: paired samples comparing VAMS versus serum sampling, and on-site versus remote VMAS sampling

Influenza virus type	Sub types	Abbreviation		VAMS versus Serum				F1 versus F2^*^			
			N	Ratio(%)^†^		SCC		Ratio(%)^‡^		SCC	
				Mean	SD	*r*	*P*_*corr*_	Mean	SD	*r*	*P*_*corr*_
A	H1	SC18	20	76.21	14.40	0.9324	<0.0001	110.09	17.41	0.8787	<0.0001
		PR8	20	78.15	14.79	0.9625	<0.0001	110.01	15.58	0.9214	<0.0001
		USSR77	20	79.46	15.56	0.9684	<0.0001	109.51	15.68	0.9423	<0.0001
		NewCal99	20	76.45	13.98	0.9532	<0.0001	110.01	16.71	0.9418	<0.0001
		Tex91	20	78.81	15.25	0.9526	<0.0001	109.49	19.42	0.9331	<0.0001
		Cali09	20	77.27	14.91	0.9542	<0.0001	110.10	16.23	0.9275	<0.0001
		Mic15	20	82.92	14.18	0.9688	<0.0001	110.39	13.43	0.9532	<0.0001
	H2	Jap57	20	87.86	22.41	0.9684	<0.0001	109.03	12.42	0.9629	<0.0001
	H3	PC73	20	84.23	14.57	0.9484	<0.0001	111.18	18.55	0.9514	<0.0001
		HK68	20	77.65	13.86	0.9685	<0.0001	109.96	15.72	0.9651	<0.0001
		Per09	20	76.95	13.93	0.9586	<0.0001	110.35	16.18	0.9701	<0.0001
		Vic11	20	77.84	14.01	0.9532	<0.0001	110.85	17.14	0.9664	<0.0001
		Tex12	20	77.65	13.23	0.9761	<0.0001	110.16	17.39	0.9703	<0.0001
		Swi13	20	74.40	14.45	0.9788	<0.0001	110.79	18.98	0.9763	<0.0001
		HK14	20	76.18	13.87	0.9792	<0.0001	110.85	17.37	0.9790	<0.0001
		Sin16	20	74.88	13.88	0.9866	<0.0001	110.62	16.05	0.9907	<0.0001
	H5	Viet04	20	81.39	16.03	0.8874	<0.0001	109.00	15.90	0.9660	<0.0001
		BJ03	20	82.20	17.97	0.9492	<0.0001	109.10	14.41	0.9571	<0.0001
	H6	chTW13	20	81.61	17.82	0.9684	<0.0001	109.89	15.40	0.9028	<0.0001
	H7	malNert00	20	80.43	16.83	0.9416	<0.0001	109.99	15.89	0.9503	<0.0001
		rheaNC93	20	95.81	30.95	0.9502	<0.0001	110.56	12.87	0.9674	<0.0001
		SH13	20	82.29	20.00	0.9501	<0.0001	112.42	15.79	0.9765	<0.0001
	H9	gfHK99	20	95.60	28.62	0.9641	<0.0001	109.96	14.31	0.9711	<0.0001
B		B/Bris08	20	84.54	21.35	0.9604	<0.0001	109.30	16.42	0.9052	<0.0001
		B/Mass12	20	86.05	14.80	0.9547	<0.0001	109.25	14.70	0.9515	<0.0001
		B/Phu13	20	96.35	19.67	0.9679	<0.0001	111.80	17.08	0.9630	<0.0001
Chimeric HA	Group 1	cH5/1	20	77.91	16.23	0.9164	<0.0001	108.44	15.34	0.9030	<0.0001
	Stalk	cH9/1	20	84.92	19.84	0.9142	<0.0001	109.41	14.85	0.9072	<0.0001
	Group 2	cH5/3	20	79.70	26.15	0.9679	<0.0001	111.65	15.59	0.9817	<0.0001
	Stalk	cH4/7	20	83.10	19.90	0.9451	<0.0001	110.12	15.25	0.9589	<0.0001

Influenza strain full names are provided in Table 1.

HA = hemagglutinin; VAMS = volumetric absorptive microsampling; SCC = Spearman’s correlation coefficient.

* F1: the finger-stick VAMS done by study coordinators on-site; F2:the finger-stick VAMS done by volunteers at-home.

† The ratio of anti-influenza virus HA IgG of VAMS sampling to that of serum sampling, expressed as a percentage (%).

‡ The ratio of anti-influenza virus HA IgG concentration F1 (on-site) to that of F2 (remote), expressed as a percentage (%).

### Adjustment of mPlex-Flu Results for Estimated Serum Volume

**Fig. 4.**
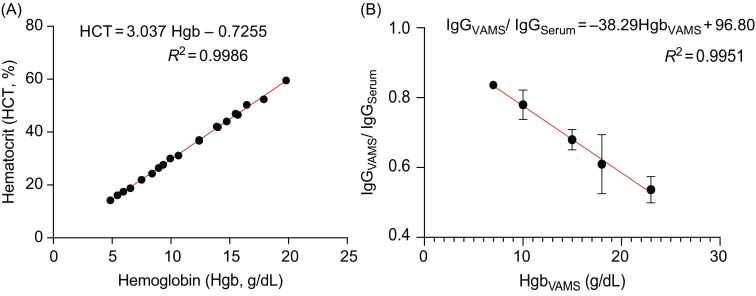
Relationship between sample Hgb, HCT, and correction of the *IgG*_*VAMS*_ concentration. (A) The correlation of HCT (determined using an automated counter) with Hgb measured from experimental whole blood samples. (B) The correlation between the ratio of anti-HA IgG from VAMS versus serum samples with Hgb concentration (*n* = 5 subjects). HA = hemagglutinin; VAMS = volumetric absorptive microsampling.

To compare the IgG concentrations measured by mPlex-Flu in VAMS versus traditional phlebotomy sampling, we calculated the ratio of mPlex-Flu values from VAMS sampling as a fraction of that from serum for each strain:(5)
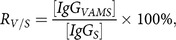
where the subscript *V* denotes a VAMS sample and *S* denotes a serum sample. We found that the mean of ratio for all anti-HA IgG measurements of *R*_*V*/*S*_ was 81.63% (Table 2), which is <1.0. This result is consistent with the fact that the denominator used to calculate [*IgG*_*S*_] is serum volume, which is a fraction the whole blood volume in the VAMS sample. This would result in lower [*IgG*_*VAMS*_] compared to [*IgG*_*S*_]. To adjust the estimate of [*IgG*_*VAMS*_] for the actual serum volume in the VAMS sample, we next estimated the serum fraction (i.e. (1 – *HCT*)) using the hemaglobin concentration of VAMS samples (see Methods). As expected, we found that Hgb is highly correlated with HCT (Fig. 4A), and that the *R*_*V*/*S*_ is inversely proportional to Hgb, as expected (Fig. 4B). These data were then used to construct a GEE for [*IgG*_*S*_] as a function of [*IgG*_*VAMS*_] and *HCT* (see Methods).

To validate this model, we calculated the estimated values [*IgG*_*ES*_] for the VAMS samples of the 20 subjects with measured [*IgG*_*VAMS*_] and [*Hgb*], then compared the estimates with the measured [*IgG*_*S*_] values. The adjusted ratios *R*_*V*/*S*_ of 30 HA influenza strain antibody concentration improved from range 74.40–95.35% (mean 81.63%) to range 82.93–109.3% (mean 90.22%) (see Table 3). [*IgG*_*ES*_] values were highly correlated with the actual [*IgG*_*S*_] values and correlation coefficients *R* are shown in Table 3. Overall, the results demonstrate that the estimated [*IgG*_*ES*_] is closer to the measured [*IgG*_*S*_] value than the uncorrected [*IgG*_*VAMS*_].

**Table 3.** VAMS and serum measurement ratio comparisons pre- and post-adjustment for hematocrit and correlations between predicted and measured serum IgG concentrations

Influenza virus type	Subtypes	Abbreviation	N	Pre-adjustment		Post-adjustment		
				R_*V/S*_(%)		R_*V/S*_(%)		SCC
				Mean	SD	Mean	SD	*r*
A	H1	SC18	20	76.21	14.40	84.42	13.10	0.8481
		PR8	20	78.15	14.79	86.42	13.17	0.9188
		USSR77	20	79.46	15.56	88.10	14.07	0.9744
		NewCall99	20	76.45	13.98	84.87	13.09	0.9444
		Tex91	20	78.81	15.25	87.39	13.04	0.9669
		Cali09	20	77.27	14.91	86.05	13.97	0.9383
		Mic15	20	82.92	14.18	92.16	12.77	0.9534
	H2	Jap57	20	87.86	22.41	96.73	19.13	0.9774
	H3	PC73	20	84.23	14.57	94.09	13.94	0.9579
		HK68	20	77.65	13.86	86.26	13.14	0.9820
		Per09	20	76.95	13.93	85.49	14.13	0.9774
		Vic11	20	77.84	14.01	86.46	13.23	0.9398
		Tex12	20	77.65	13.23	85.94	13.29	0.9669
		Swi13	20	74.40	14.45	82.61	13.71	0.9459
		HK14	20	76.18	13.87	84.48	13.19	0.9549
		Sin16	20	74.88	13.88	82.93	12.73	0.9744
	H5	Viet04	20	81.39	16.03	90.88	14.41	0.9489
		BJ03	20	82.20	17.97	89.79	16.23	0.9504
	H6	chTW13	20	81.61	17.82	90.16	15.09	0.9323
	H7	malNert00	20	80.43	16.83	88.43	15.12	0.9278
		rheaNC93	20	95.81	30.95	103.89	28.55	0.9579
		SH13	20	82.29	20.00	90.03	17.16	0.9143
	H9	gfHK99	20	95.60	28.62	105.37	27.75	0.9429
B		B/Bris08	20	84.54	21.35	94.12	19.94	0.9098
		B/Mass12	20	86.05	14.80	95.24	13.05	0.9729
		B/Phu13	20	96.35	19.67	107.4	20.63	0.9549
Chimeric HA	Group 1	cH5/1	20	77.91	16.23	85.39	13.93	0.9278
	Stalk	cH9/1	20	84.92	19.84	93.79	19.08	0.9128
	Group 2	cH5/3	20	79.70	26.15	86.75	22.13	0.9068
	Stalk	cH4/7	20	83.10	19.90	90.97	17.85	0.8737
			Average	81.63		90.22		

Influenza strain full names are provided in Table 1.

HA = hemagglutinin; VAMS = volumetric absorptive microsampling; SCC = Spearman’s correlation coefficient.

*R*_*V*/*S*_ = the ratio of anti-influenza virus HA IgG concentration of VAMS sampling to that of serum sampling, expressed as a percentage (%).

### VAMS is a Highly Reproducible Method When Performed at-Home

One advantage of the VAMS method is the safety and simplicity of the process. It is easy for study volunteers to learn and perform at-home. Previously published data have shown that the volume of blood captured in the 10 µL VAMS device varies <0.4 µL [17]. But no study has shown the reputability of VAMS sampling by participants at-home compared to on-site by a nurse in the mPlex-Flu assay to evaluate influenza virus antibodies. To estimate this correlation, a second finger-stick collection was performed by the 20 subjects at-home 3 days later. These samples were then hand-delivered back to the laboratory in a provided envelope.

Using the same analytic approach, we calculated the correlation of at-home (*F*2) and on-site (*F*1) sampling for measurement of *IgG*-mediated immunity across multiple influenza virus strains, grouped by strain and subject and adjusted using simultaneous Hgb measurements. The results are shown in Table 2, and Figs. 3C and 3D. We found no statistically significant difference between the results obtained with on-site versus at-home VAMS sampling. These data suggest that VAMS sampling could be preformed at-home by the study subjects, as the anti-HA antibody concentrations are highly consistent with sampling performed by study personnel on-site. These results support the consistency of VAMS sampling for future influenza vaccine or infection immunity studies.

### Stability of Anti-Influenza HA Antibodies in VAMS Samples

**Fig. 5.**
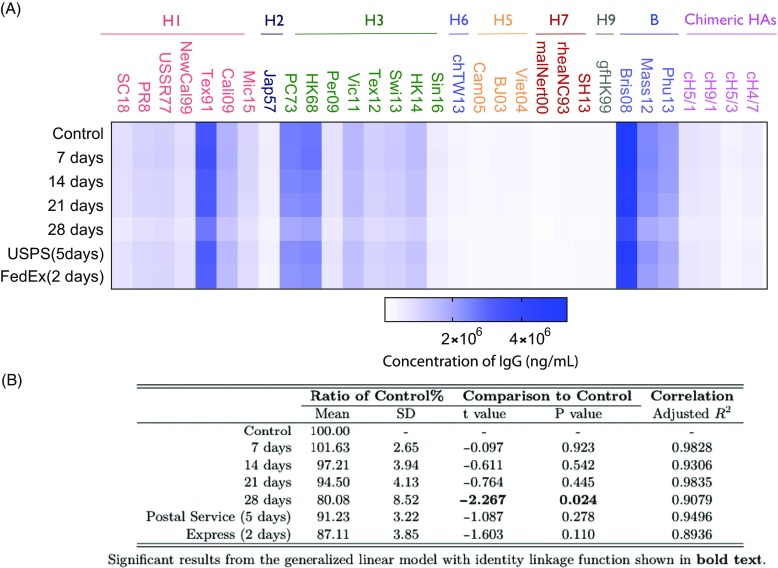
The stability of multiple dimensional IgG antibodies collected with VAMS finger stick stored at room temperature or after shipping. The mean concentration of influenza virus IgG antibodies against 30 strains of influenza virus HA by mPlex-Flu assay is shown in the heatmap (*n* = 4). USPS = United States Postal Service; FEDEX = Federal Express Overnight Shipping; SD = Standard Deviation; HA = hemagglutinin; VAMS = volumetric absorptive microsampling. Influenza strain full names are provided in Table 1.

We next examined the stability of anti-influenza virus HA antibodies in samples stored in VAMS device at room temperature, and during transport (e.g. postal service, 2-day express mail). This is an essential aspect of quality control that needs to be addressed for future applications of VAMS. Prior studies have shown that antibodies on DBS filter paper are stable for more than 20 years when stored at 4 or −20°C [16]. To determine the stability of antibodies in the VAMS device over time at room temperature, we used the mPlex-Flu assay to compare the antibody activity of VAMS tips stored at −20°C immediately after drying (control) with other VAMS tips left for 7, 14, 21, or 28 days at room temperature. The results are shown in Fig. 5A. We found no detectable antibody activity decrease at room temperature from storing the VAMS devices at room temperature environment, and antibody measurements still kept 94.5% of the control level up to 21 days. After storing VAMS devices at room temperature to 28 days, the antibody activity level was significantly decreased to 80% of the control levels from the control devices stored at −20°C (statistical results were shown in the table in Fig. 5B).

To confirm the stability of the antibodies during the shipping process, we compared two commonly used shipping methods: standard first-class mail and 2-day commercial shipping (Federal Express, FedEx) to send two duplicate groups of the VAMS devices to our lab in New York State in August. USPS first-class mail took 5 days and commercial shipping took 2 days. After the samples were received back at the lab, the anti-HA IgG antibody levels were evaluated by mPlex-Flu assay (Fig. 5A). No statistically significant difference was detected between results from samples transported via the two shipping methods (Fig. 5B), suggesting that the VAMS samples are stable during shipping process (2–5 days) even during the summer time, when temperatures may be elevated.

## Discussion

In this report, we have demonstrated the utility of capillary VAMS sampling, combined with the mPlex-Flu assay, for measuring anti-influenza HA IgG antibody levels. This combination addresses a significant translational barrier in influenza research and population health research: how to measure antibody-mediated influenza immunity in a large number of subjects at modest expense using a minimally invasive method for sample collection. The VAMS sampling method is inexpensive, can be used remotely by study volunteers, requires only a finger stick, and yields consistent results compared to serum samples obtained with standard phlebotomy. VAMS samples are stable at room temperature for up to 21 days, and during standard 2–5 day shipping at ambient temperatures. Combined with the mPlex-Flu method, we are able to measure IgG reactivity against more than 30 influenza strains simultaneously from a 10-µL VAMS finger-stick sample [8].

We have also demonstrated that the VAMS-mPlex Flu method overcomes an issue with the use of traditional DBS methods for estimating the concentration of analytes distributed only in the serum compartment [30], such as IgG. Serum typically accounts for ∼45–80% of total blood volume, and thus whole blood concentrations of IgG will be lower than serum estimates, and vary by test subject HCT. This could lead to underestimation of vaccine infection-induced changes in IgG in clinical trials or research studies. We have shown here that simultaneous measurement of Hgb content in VAMS samples can be used to estimate serum volume and to improve estimates of serum [*IgG*]. One minor caveat is that even with this adjustment, the ratio of IgG concentrations of samples obtained by VAMS versus serum was still *R*_*V*/*S*_ ≠ 1.0. This could be due to a variety of factors, including variation in microcapillary sampling by gender, Hgb retained on the VAMS device out of proportion to IgG, or other factors. Thus, further work could be done to improve the estimating equations in larger population studies. Nevertheless, the improved correlation and prediction of [*IgG*_*S*_] from [*IgG*_*V*_] suggests that this relatively simple method may be adequate for many studies.

It is also important to note that serum volume correction may not be needed when comparing the ratio of pre- to post-vaccine anti-HA IgG concentrations over a short time period, and in the same subject, when plasma volume is unlikely to change significantly. This is likely the case for measurements within 30 days of vaccination, which constitute a large fraction of the samples collected in current influenza vaccine research studies. However, conditions in which the red cell volume is increased (erythrocytosis) or markedly decreased (sickle cell disease, *β* -thallasemia, blood loss anemia) may affect between subject comparisons [19,31]. Thus studies with large population comparisons between subjects, and over long time periods in diverse populations, may benefit from this adjustment to avoid underestimating absolute anti-HA IgG concentrations. Finally, whether such differences in how IgG concentrations are expressed have clinical relevance in vaccine immunity studies is currently unclear.

We suggest that this method may improve future studies of the longitudinal persistence of IgG-mediated influenza immunity. It is known that IgG-mediated influenza immunity can decrease after vaccination over months, but vaccine trials and research studies rarely sample beyond 90 days post-vaccination [22]. A major translational issue in vaccine trials is the expense and difficulty of having study subjects come to a study center for phlebotomy to monitor vaccine responses. A combined VAMS + mPlex-Flu methodology could allow for longer term remote sample collection from study subjects, along with assay of IgG-mediated anti-influenza antibody levels. This would greatly lower the cost and difficulty of such monitoring, and aid in current research initiatives to develop a universal influenza vaccine [32], to understand the persistence of vaccine-mediated immunity [33], and to predict who will respond to influenza vaccines long term [34].

The combination of VAMS sampling and mPlex-Flu analysis also has the potential to reduce translational barriers to large-scale population studies of antibody-mediated influenza immunity [2,24,35]. This method would enable remote subject enrollment, consent, and sample collection across large populations and disparate geographic areas. In addition, this method also addresses a significant translational barrier to determining the true efficacy of the seasonal influenza vaccine, which is based on data collected by influenza surveillance field teams. Currently, WHO and CDC field surveillance teams only collect vaccine history and swabs for influenza strain genotyping. Providing WHO and CDC teams with VAMS collection kits when surveilling influenza-like illness cases in the field, especially in remote areas, would allow public health agencies to determine if infected subjects actually had, or lacked, antibody-mediated immunity against the circulating influenza strains [36]. This is not currently done on any large scale and would provide much more accurate information regarding vaccine efficacy. Finally, the mPlex-Flu assay provides rich, multidimensional data regarding IgG anti-HA reactivity. Informatics methods can be used to calculate the antigenic distance between different influenza strain HAs using sequence comparisons. When combined with anti-HA IgG levels from the study of very large populations, such data could be used to create large-scale HA antigenic landscapes [37,38] for future influenza immunity research.

Finally, we also would like to note that this approach is not pathogen-specific, but could be adapted to estimating antibody-mediated immunity after vaccination for any other viral pathogens, where the key antibody targets are known and broad populations need to be surveyed. In addition, other biologic data could easily be obtained from a second VAMS device (another 10 µL of blood), including DNA samples for genomic sequencing of the host or blood-borne pathogens. Such methods are likely to be increasingly used in clinical trials in remote and rural areas, as well as newer “siteless” clinical trials [39,40].

## Data Availability

The primary data generated by this study are available from https://figshare.com/s/7f8992762e960e0aa023

## Supplementary material

For supplementary material accompanying this paper visit https://doi.org/10.1017/cts.2019.410. click here to view supplementary material
