# Supplementary material for: Application of volumetric absorptive microsampling (VAMS) to measure multidimensional anti-influenza IgG antibodies by the mPlex-Flu assay
Source: J Clin Transl Sci. 2019 Sep 26;3(6):332–43. doi: 10.1017/cts.2019.410 (PMC6885997; doi:10.1017/cts.2019.410)
Supplement: Supplementary file 1 [file S2059866119004102sup001.pdf]

Supplementary Data:

Subject demographics

Twenty-one healthy volunteers were recruited for this study and their demographics are shown in Supplementary Table. More female subjects took part in this study (71%) than male ($p = 0.0784$), with majority of volunteers being Caucasian (90%; $p = 0.002$). The distribution of age groups is relatively uniform with fewer volunteers $\leq$ 20 years of age.

Table. Subject Demographics

	**Summary**	**N(%)**
Sex	Female	15(71)
	Male	6 (29)
Ethnicity	Hispanic / Latino	0 (0)
	White	19 (90)
	Black or African American	0 (0)
	Asian	2 (10)
	Native American / Alaska Native	0 (0)
	Native Hawaiian / Pacific Islander	0 (0)
Age	<20	2 (10)
	21-45	7 (33)
	46-60	7 (33)
	61-75	5 (24)
Total		21(100)
